# Reaction‐Superdiffusion Systems in Epidemiology, an Application of Fractional Calculus

**DOI:** 10.1063/1.3241397

**Published:** 2009-09-09

**Authors:** Nico Stollenwerk, João Pedro Boto

**Affiliations:** Centro de Matemática e Aplicações Fundamentais CMAF, Universidade de Lisboa, Avenida Prof. Gama Pinto 2, 1649‐003 Lisboa, Portugal; University of Peloponnese; University of Buckingham; Technological Educational Institution of Chalkis

## Abstract

Spatially extended stochastic processes in epidemiology lead to classical reaction‐diffusion process, when infection spreads only locally. This notion can be generalized using fractional derivatives, especially fractional Laplacian operators, leading to Lévy flights and sub‐ or super‐diffusion. Especially super‐diffusion is a more realistic mechanism of spreading epidemics than ordinary diffusion.

## INTRODUCTION

Classical derivatives of integer order have been generalized historically in various ways to derivatives of fractional order [1, 2] and especially [3, 4] with more reference and results. Especially, the Weyl-derivative generalizing the derivative in Fourier space is of interest here. It is linked with the Riemann-Liouville via the Marchaud regularization [2]. In this way the Laplacian operator can be generalized to a fractional Laplacian describing Lévy flights rather than ordinary random walks. This leads to the notion of sub- and super-diffusion, well applicable in reaction-diffusion systems [6]. In epidemiological systems especially the super-diffusion case is of interest as description of more realistic spreading than normal diffusion on regular lattices. To understand even basic epidemiological processes it is often necessary to investigate well the spatial spreading since all epidemic processes happen on spatially restricted networks [7]. We have previously studied epidemic processes with reinfection on regular lattices [13] as they also appear in the physics literature [10]. A crucial question in such systems is in how far basic notions like finite spreading and phase diagrams hold not only for ordinary diffusion but also in the super-diffusion case [7, 8]. Wider processes with multi strain interaction [12, 5] could be treated similarly. As our prime example here we will investigate the susceptible-infected- susceptible SIS epidemic, which leads in the framework of reaction diffusion processes to the well know Kolmogorov-Fisher equation [11, 9].

## FROM STOCHASTIC EPIDEMIC MODELS TO REACTION-DIFFUSION PROCESSES

The SIS epidemics is an autocatalytic process given by the reaction scheme

$$\begin{aligned} S+I &\xrightarrow{\beta} I+I \\ I &\xrightarrow{\alpha} S \end{aligned}$$

and can be described via a master equation to capture the population noise of the epidemiological model (see [14] for a more detailed description of the SIS process). The stochastic spatially extended SIS epidemic process on general lattice or network topologies is given by the following dynamics for the probability $p$ of the state of a network

$$\begin{aligned} \frac{d}{dt}p(I_1,I_2,...,I_N,t) &= \sum_{i=1}^{N}\beta\left(\sum_{j=1}^{N}J_{ij}I_j\right)I_i\ p(I_1,...,1-I_i,...,I_N,t)+\sum_{i=1}^{N}\alpha(1-I_i)\ p(I_1,...,1-I_i,...,I_N,t) \\ &-\sum_{i=1}^{N}\left[\beta\left(\sum_{j=1}^{N}J_{ij}I_j\right)(1-I_i)+\alpha I_i\right]\ p(I_1,...,I_i,...,I_N,t) \end{aligned} \quad (1)$$

CP1168, Vol. 2, *Numerical Analysis and Applied Mathematics, International Conference 2009*
edited by T. E. Simos, G. Psihoyios, and Ch. Tsitouras
© 2009 American Institute of Physics 978-0-7354-0708-4/09/$25.00

for variables $I_i \in \{0,1\}$ and adjacency matrix $(J_{ij})$. Local quantities like the expectation value of infected at a single lattice point, which in reaction diffusion systems corresponds to the local density $u(x,t)$ are given by

$$\langle I_i\rangle(t) := \sum_{I_1=0}^{1}\sum_{I_2=0}^{1}\dots\sum_{I_N=0}^{1} I_i\, p(I_1,I_2,...,I_N,t) \quad . \tag{2}$$

For such quantities dynamics can be derived using the original dynamics of the stochastic process description for $p(I_1,I_2,...,I_N,t)$. In such dynamics for local quantities there appears the discretized diffusion operator in the case of lattice models

$$\Delta\langle I_i\rangle := \sum_{j=1}^{N} J_{ij}(\langle I_j\rangle - \langle I_i\rangle) \tag{3}$$

and defines a generalized Laplace-operators for other network topologies, coded in the adjacency matrix $(J_{ij})$. Considering the local quantity $\langle I_i\rangle(t)$, which in a countinuous space model corresponds to the local density $u(x,t)$ with spatial variable $x$ corresponding to $i$ and lattice spacing $a$ from our lattice model going to zero, we obtain

$$\frac{d}{dt}\langle I_i\rangle = \beta\sum_{j=1}^{N} J_{ij}\langle(1-I_i)I_j\rangle - \alpha\langle I_i\rangle \quad . \tag{4}$$

Hence

$$\frac{d}{dt}\langle I_i\rangle \;=\; \beta\sum_{j=1}^{N} J_{ij}(\langle I_j\rangle - \langle I_i\rangle) + \beta\sum_{j=1}^{N} J_{ij}\langle I_i\rangle - \beta\sum_{j=1}^{N} J_{ij}\langle I_i I_j\rangle - \alpha\langle I_i\rangle \tag{5}$$

where we now use the discrete version of the diffusion operator $\Delta\langle I_i\rangle = \sum_{j=1}^{N} J_{ij}(\langle I_j\rangle - \langle I_i\rangle)$ for the first term of the sum on the right hand side of the equation. Further, in the term $-\beta\sum_{j=1}^{N} J_{ij}\langle I_i I_j\rangle$ we apply a local mean field assumption in the sense that local correlations can be neglected and coarse grained hence $\langle I_i I_j\rangle - \langle I_i\rangle\langle I_j\rangle \approx 0$ and $\langle I_i\rangle\langle I_j\rangle \approx \langle I_i\rangle\langle I_i\rangle$. Furthermore, we use $Q_i = \sum_{j=1}^{N} J_{ij}$ for the total number of neighbours of lattice site $i$, and in regular lattices $Q_i = Q$ as a single constant for the number of neighbours of any lattice site. Hence we finally obtain

$$\frac{d}{dt}\langle I_i\rangle = \beta Q\langle I_i\rangle\left(1-\langle I_i\rangle\right) - \alpha\langle I_i\rangle + \beta\Delta\langle I_i\rangle \quad . \tag{6}$$

This is for lattice spacing going to zero, hence $u(x,t) = \langle I_i\rangle$ nothing but the Kolmogorov-Fisher equation in the form

$$\frac{\partial}{\partial t}u = r\,u\left(1-\frac{u}{k}\right) + \chi\Delta u \tag{7}$$

where we identify the growth rate $r = \beta Q - \alpha$, the carrying capacity $k = \left(1-\frac{\alpha}{\beta Q}\right)$ and diffusion constant $\chi = \beta$. Often the carrying capacity is simply set to unity, as well as the diffusion constant.

## FRACTIONAL CALCULUS: FROM DIFFUSION TO SUPER-DIFFUSION

Here we concentrate on the diffusion part of Eq. (7), hence we look at a Fokker-Planck type equation for simple diffusion, which also describes a random walker in space (see Fig. 1 a), there for graphical reasons in two spatial dimensions)

$$\frac{\partial}{\partial t}\; p(x,t|x_0,t_0) = \frac{1}{2}\frac{\partial^2}{\partial x^2}\; p(x,t|x_0,t_0) \tag{8}$$

with the solution

$$p(x,t|x_0,t_0) = \frac{1}{\sqrt{2\pi(t-t_0)}}\; e^{-\frac{(x-x_0)^2}{2(t-t_0)}} \tag{9}$$

which is a Gaussian distribution with mean value $\mu = x_0$ and time dependent variance $\sigma^2 = (t-t_0)$. The mean displacement $\sqrt{\langle(x-x_0)^2\rangle}$ has the famous $\sqrt{t}$ behaviour.

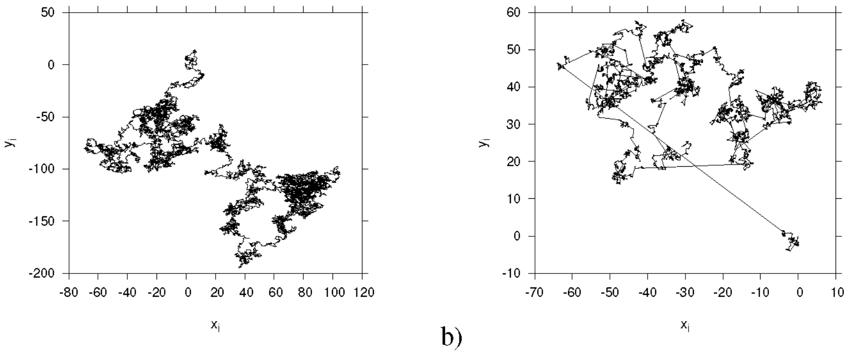

a) b) .

**FIGURE 1.** a) Random walk with Gaussian distributed steps. b) Lévy flight with exponent $\mu = 1.5$

The Fourier transform of the probability $p(x,t) := p(x,t|x_0 = 0, t_0 = 0)$ of the Wiener process, Eq. (9), is simply

$$\tilde{p}(k,t) = e^{-k^2 t} \tag{10}$$

and the Fokker-Planck equation is in Fourier space given by

$$\frac{\partial}{\partial t} \tilde{p}(k,t) = -k^2 \cdot \tilde{p}(k,t) \tag{11}$$

which now can be easily generalized to other powers of $k$ than the power of 2 for normal diffusion.

Fractional calculus, i.e. the generalization of first, second etc. derivatives to derivatives of non-integer order, hence fractional, gives us the tools to generalize also the Laplace operator in diffusion systems. Historically, many ways of generalizing integer derivatives have been suggested. Here we use the generalization in terms of the Fourier expansion of a function, hence the Weyl-derivative, which is linked via the Marchaud-regularization to the Riemann-Liouville-derivative. Hence, to describe super-diffusion we generalize the Fourier representation of the diffusion process to $\mu \in (0,2]$ in the solution

$$\tilde{p}(k,t) = e^{-|k|^{\mu} t} \tag{12}$$

which corresponds to

$$\frac{\partial}{\partial t} \tilde{p}(k,t) = -|k|^{\mu} \cdot \tilde{p}(k,t) \tag{13}$$

in the Fokker-Planck equation. By inverse Fourier transformation we obtain in real space representation

$$\frac{\partial}{\partial t} p(x,t) = -\left(-\Delta_x\right)^{\mu/2} p(x,t) \quad . \tag{14}$$

For $\mu \in (0,1)$ the fractional Laplacian operator $(-\Delta_x)^{\mu/2}$ is given by

$$(-\Delta_x)^{\mu/2} p(x,t) = C_\mu \int_{-\infty}^{\infty} \frac{p(x,t) - p(y,t)}{|x-y|^{1+\mu}} \, dy \tag{15}$$

with constant $C_\mu = \left( \frac{2^{-\mu} \pi^{3/2}}{\Gamma(1+\frac{\mu}{2}) \Gamma(\frac{1+\mu}{2}) \sin(\frac{\mu\pi}{2})} \right)^{-1}$. For $\mu \in [1,2)$ the fractional Laplacian operator $(-\Delta_x)^{\mu/2}$ is given by

$$(-\Delta_x)^{\mu/2} p(x,t) = C_\mu \int_{-\infty}^{\infty} \frac{\triangle_{x-y} [p(x,t) - p(y,t)]}{|x-y|^{1+\mu}} \, dy \tag{16}$$

where $\triangle_{x-y}$ is the central-difference operator,

$$\begin{aligned} \triangle_{x-y} [p(x,t) - p(y,t)] &= p(y,t) - p(x,t) - (p(y-(y-x),t) - p(x-(y-x),t)) \\ &= p(y,t) - 2p(x,t) + p(2x-y,t) \quad , \end{aligned} \tag{17}$$

with constant $C_\mu = \left( \frac{-(2^{1-\mu})\pi^{3/2}}{\Gamma(1+\frac{\mu}{2})\Gamma(\frac{1+\mu}{2})\sin(\frac{\mu\pi}{2})} \right)^{-1}$. Or as master equation it can be written

$$\frac{\partial}{\partial t}\ p(x,t) = \int w_{x|y} p(y,t) - w_{y|x} p(x,t)\ dy \tag{18}$$

with transition rate $w_{x|y} = \frac{C_\mu}{|x-y|^{1+\mu}}$ for $\mu \in (0,1)$ and $w_{x|y} = \frac{C_\mu}{|x-y|^{1+\mu}} \triangle_{x-y}$ for $\mu \in [1,2)$. The solution in real space representation for $t > t_0$ is given by

$$p(x,t|x_0,t_0) = \frac{1}{2\pi} \int e^{-ik(x-x_0)-|k|^\mu (t-t_0)}\ dk \tag{19}$$

or with the function $G_\mu(z) := \frac{1}{2\pi} \int e^{-ikz-|k|^\mu}\ dk$ the solution is

$$p(x,t|x_0,t_0) = \frac{1}{(t-t_0)^{1/\mu}}\ G\left( \frac{x-x_0}{(t-t_0)^{1/\mu}} \right) \quad . \tag{20}$$

The function $G_\mu(z)$ has for large argument $|z| >> 1$ a power law tail

$$G_\mu(z) \sim \frac{1}{|z|^{1+\mu}} \quad . \tag{21}$$

An example for a process with long range jumps we plot in Fig. 1 b) a Lévy flight with exponent $\mu = 1.5$, again as in a) for graphical reasons in two spatial dimensions.

## ACKNOWLEDGMENTS

We thank Luis Sanchez, Jose Francisco Rodrigues and Stanislav Antontsev, Lisbon, Dirk Brockmann, Göttingen and Chicago, and Frank Hilker, Lisbon and Bath, for stimulating discussions on the present topic. We further thank FCT, Portugal, and the EU Framework Program 7 under the project EPIWORK for financial support.
